# Complete sequences of epidermin and nukacin encoding plasmids from oral-derived *Staphylococcus epidermidis* and their antibacterial activity

**DOI:** 10.1371/journal.pone.0258283

**Published:** 2022-01-18

**Authors:** Kenta Nakazono, Mi Nguyen-Tra Le, Miki Kawada-Matsuo, Noy Kimheang, Junzo Hisatsune, Yuichi Oogai, Masanobu Nakata, Norifumi Nakamura, Motoyuki Sugai, Hitoshi Komatsuzawa

**Affiliations:** 1 Department of Oral and Maxillofacial surgery, Field of Maxillofacial Rehabilitation, Kagoshima University Graduate School of Medical and Dental Sciences, Kagoshima, Japan; 2 Department of Bacteriology, Hiroshima University Graduate School of Biomedical and Health Sciences, Hiroshima, Japan; 3 Project Research Centre for Nosocomial Infectious Diseases, Hiroshima University, Hiroshima, Japan; 4 Antimicrobial Resistance Research Center, National Institute of Infectious Diseases, Higashi Murayama, Japan; 5 Department of Oral Microbiology, Kagoshima University Graduate School of Medical and Dental Sciences, Kagoshima, Japan; College of Medicine, National Taiwan University, TAIWAN

## Abstract

*Staphylococcus epidermidis* is a commensal bacterium in humans. To persist in the bacterial flora of the host, some bacteria produce antibacterial factors such as the antimicrobial peptides known as bacteriocins. In this study, we tried to isolate bacteriocin-producing *S*. *epidermidis* strains. Among 150 *S*. *epidermidis* isolates from the oral cavities of 287 volunteers, we detected two bacteriocin-producing strains, KSE56 and KSE650. Complete genome sequences of the two strains confirmed that they carried the epidermin-harboring plasmid pEpi56 and the nukacin IVK45-like-harboring plasmid pNuk650. The amino acid sequence of epidermin from KSE56 was identical to the previously reported sequence, but the epidermin synthesis-related genes were partially different. The prepeptide amino acid sequences of nukacin KSE650 and nukacin IVK45 showed one mismatch, but both mature peptides were entirely similar. pNuk650 was larger and had an additional seven ORFs compared to pIVK45. We then investigated the antibacterial activity of the two strains against several skin and oral bacteria and found their different activity patterns. In conclusion, we report the complete sequences of 2 plasmids coding for bacteriocins from *S*. *epidermidis*, which were partially different from those previously reported. Furthermore, this is the first report to show the complete sequence of an epidermin-carrying plasmid, pEpi56.

## Introduction

Staphylococci are classified into two groups, *Staphylococcus aureus* and coagulase -negative staphylococci (CoNS) due to their clinical importance. CoNS are abundant colonizers on the skin and are considered to contribute to the maintenance of skin integrity and homeostasis [1–3]. CoNS assist in immune activity to prevent pathogen colonization by inducing antimicrobial peptides from the epithelium, by direct production of antibacterial factors such as phenol-soluble modulins (PSMs) and bacteriocins [4–6]. Therefore, the colonization of CoNS provides several benefits to the host. However, CoNS are commonly isolated in clinical cultures and considered to be major nosocomial pathogens in humans [7, 8]. CoNS are often isolated from blood and indwelling medical implants such as intravascular catheters and urinary catheters, leading to opportunistic infectious diseases. In addition, most clinical isolates of *Staphylococcus epidermidis* carry the genes encoding for antibiotic resistance and biofilm formation, which significantly challenge current antibiotic therapy [9, 10].

In the oral cavity, oral bacterial flora is composed of a great diversity of bacterial species. Many oral indigenous bacteria, including oral streptococci, are known to produce antimicrobial factors such as bacteriocins and hydrogen peroxide [11–15]. Bacteriocins exhibit a wide range of antimicrobial activity against the bacterial species that make up the oral flora [16]. Therefore, bacteriocins are thought to be involved in the exclusion and symbiosis of other bacteria in the oral cavity. *S*. *epidermidis* is also found in oral cavity [17, 18]. Some *S*. *epidermidis* are known to produce antimicrobial peptides known as bacteriocins, including epidermin [19–21], Pep5 [21–23], epilancin K7 [21, 24], epilancin 15X [25, 26], epicidin 280 [27] and Nukacin IVK-45 [28]. These bacteriocins are known to be lantibiotics containing specific amino acids such as lanthionine, β-methyllanthionine, and dehydrated amino acids [11–13]. However, there are no reports about the bacteriocin produced by *S*. *epidermidis* isolated from the oral cavity.

So far, there have been many reports on bacteriocins produced by oral isolates of streptococcal species [11–13, 16] but very few reports on other oral bacterial species. To understand the meaning of bacteriocins for bacterial flora formation, more information about bacteriocins produced by many oral bacterial species is required. In this study, we focused on the bacteriocins of oral-derived *S*. *epidermidis* to understand the antibacterial activity against oral and skin bacteria. We examined 150 *S*. *epidermidis* strains isolated from the oral cavity and investigated their bacteriocin-producing activity. As a result, we found two strains that produced epidermin and nukacin IVK45. We performed the complete-genome analysis of these two strains and identified the plasmids harboring the epidermin or nukacin IVK45-like bacteriocin gene clusters. The nucleotide sequences of these plasmids were not entirely similar to the previously reported sequences. Additionally, we evaluated the antibacterial activity of these two bacteriocins against the skin and oral commensal bacteria.

## Materials and methods

### Bacterial strains and growth conditions

*S*. *epidermidis* clinical isolates were grown in trypticase soy broth (TSB) (Becton, Dickinson and Company [BD], Franklin Lakes, NJ, USA) at 37°C. The *Staphylococcus aureus* MW2 strain and *braRS*-inactivated mutant were obtained previously [29]. Other bacteria used in this study are listed in Table 1. Staphylococcal strains and *Micrococcus luteus* were grown in TSB at 37°C and 30°C, respectively. Streptococcal strains were grown in TSB at 37°C with 5% CO2. *Cutibacterium acnes* was grown on sheep blood agar at 37°C anaerobically. *Corynebacterium* and *Rothia mucilanginosa* were grown at 37°C in R medium and BHI (BD) aerobically, respectively. The composition of R medium is as follows: 1g of bacto peptone (BD), 0.5g of yeast extract (BD), 0.5g of malt extract (BD), 0.5g of casamino acids (BD), 0.2g of beef extract (BD), 0.2g of glycerol, 5mg of Tween 80, 0.1g of MgSO_4_ in 100 ml distilled water. When necessary, tetracycline (5 μg/ml) was added to the medium.

**Table 1:** Strains used in this study.

Strains	Character	origin
*Staphylococcus epidermidis*		
KSE1	Wild type	This study
KSE3	Wild type	This study
KSE56	Wild type	This study
KSE650	Wild type	This study
KSE56-	KSE56 plasmid deleted	This study
KSE650-	KSE650 plasmid deleted	This study
*Staphylococcus warneri* ISK-1	Wild type	
*Staphylococcus hominis* JCM31912	Wild type	Riken BRC ^1^
*Staphylococcus haemolyticus* JCM2416	Wild type	Riken BRC ^1^
*Staphylococcus capitis* subsp. capitis JCM2420	Wild type	Riken BRC ^1^
*Staphylococcus simulans* JCM2424	Wild type	Riken BRC ^1^
*Cutibacterium acnes* JCM6425	Wild type	Riken BRC ^1^
*Corynebacterium accolens* JCM8331	Wild type	Riken BRC ^1^
*Corynebacterium pseudodiphtheriticum* JCM1320	Wild type	Riken BRC ^1^
*Rothia mucilaginosa* JCM10910	Wild type	Riken BRC ^1^
*Micrococcus luteus* JCM1464	Wild type	Riken BRC ^1^
*Streptococcus mutans* UA159	Wild type	[30]
*Streptococcus sanguinis* GTC217	Wild type	Gifu University
*Streptococcus salivarius* GTC215	Wild type	Gifu University
*Streptococcus gordonii* JCM12995	Wild type	Riken BRC ^1^
*Staphylococcus aureus*		
COL	Wild type	[31]
RN4220	NCTS8325 derivative	[32]
MW2	clinical strain, methicillin-resistant (*mecA*+)	[33]
ΔTCS16	*braRS* inactivation in MW2, Tcr2	[29]

1. Japan Collection of Microorganisms.

2. Tetracycline resistance.

### Isolation of *Staphylococcus epidermidis* from the oral cavity

*S*. *epidermidis* strains were isolated from the oral cavities of 287 volunteers. Saliva collected from the oral cavity was plated on No.110 medium (Eiken Chemical Co. Ltd, Tokyo, Japan) and incubated for 2 days at 37°C. The strains were picked from a single white colony on the agar and further investigated by PCR with specific primers for *S*. *epidermidis* (forward primer: GGCAAATTTGTGGGTCAAGA, reverse primer: TGGCTAATGGTTTGTCACCA). Isolated *S*. *epidermidis* strains were replated on TSB containing 2% agar (TSA) medium. The isolated strains were then replated again on TSA to pick up a single colony and finally, *S*. *epidermidis* confirmed by PCR was used in this study. Clinical isolates were designated as KSE strains. Saliva collection and *S*. *epidermidis* isolation were approved by the Ethical Committee of the Kagoshima University Graduate School of Medical and Dental Sciences (No. 701) and the Ethical Committee for Epidemiology of Hiroshima University (E-1998). Written informed consent was obtained from all participants. All methods were performed in accordance with the approved guidelines and regulations.

### Screening of bacteriocin-producing *S*. *epidermidis*

To investigate bacteriocin production among *S*. *epidermidis* strains, we performed a direct assay using *S*. *aureus* MW2 *braRS* knockout mutant as an indicator strain because this mutant showed increased susceptibility to several bacteriocins [34]. Overnight cultures of *S*. *epidermidis* strains were spotted on a TSA plate and cultured at 37°C for 24 h. Then, 3.5 ml of prewarmed half-strength TSB soft agar (1%) containing *braRS* knockout mutant cells (10^7^ cells/ml) were poured over the TSA plate. The plates were incubated at 37°C for 24 h. The strains which showed the growth inhibition zones surrounding *S*. *epidermidis* strain were picked up. The strains were reconfirmed for bacteriocin production by the direct assay again.

### Complete genome sequences of bacteriocin-producing *S*. *epidermidis* strains

To perform whole-genome sequencing of *S*. *epidermidis* strains, DNA was extracted from each strain. *S*. *epidermidis* cells grown overnight in 5 ml TSB were collected and then suspended in 0.5 ml of CS buffer (100 mM Tris-HCl [pH 7.5], 150 mM NaCl, 10 mM EDTA) containing lysostaphin (Sigma-Aldrich, St. Louis, MO, USA) (final concentration: 50 μg/ml) and RNase (Nippon Gene, Tokyo, Japan) (final: 20 μg/ml). After incubation at 37°C for 1 h, proteinase K (Nacalai Tesque, Kyoto, Japan) (final: 150 μg/ml) and SDS (final 1%) were added, followed by incubation at 55°C for 5 h. After treatment with phenol followed by phenol-chloroform, DNA was precipitated by ethanol. Whole-genome sequencing (WGS) of *S*. *epidermidis* strains **w**as performed using the Illumina MiSeq sequencing platform, followed by annotation with Rapid Annotation using Subsystem Technology (RAST) version 2.0 [35]. After confirming the presence of bacteriocin genes using WGS, long-read sequencing by MinION (Oxford Nanopore Technologies, UK) was carried out to determine the complete sequences of the chromosomes and plasmids of these strains. Hybrid assembly of Illumina short reads and MinION long reads was performed with Unicycler v0.4.8. The complete sequences of plasmids harboring bacteriocin genes were selected, including epidermin-carrying plasmid pEpi56 and nukacin-carrying plasmid pNuk650. Each plasmid was compared with publicly available plasmids or gene clusters, including the *epiY’-epiP* gene cluster (X62386), *epiG-epiT’’* gene cluster (U77778), and pIVK45 (accession number KP702950).

### Accession numbers

The complete plasmids carrying epidermin (pEpi56) and nukacin (pNuk650) have been deposited in the NCBI database under accession numbers OK031036 and OK031035, respectively.

### Identification of epidermin and nukacin KSE650 produced by *S*. *epidermidis*

To identify the bacteriocin, we purified the bacteriocin from two *S*. *epidermidis* strains. Overnight cultures (500 ml) of *S*. *epidermidis* KSE56 and KSE650 were centrifuged at 4,000 x g for 15 min. Macro-Prep cationic resin (1.5 ml) (Bio rad, USA) was added to the supernatant and stirred for 12 h. The resin was collected into an open column, then washed three times with 10 ml of 25 mM ammonium acetate (pH 7.5). To elute the bacteriocin, the resin was treated with 500 μl of 5% acetic acid. This elution was repeated 10 times. After each fraction was evaporated completely, the samples were dissolved in 50 μl of distilled water. Each solution was tested for antibacterial activity against *M*. *luteus*. Overnight cultures of *M*. *luteus* (100 μl) were inoculated on TSA plates. Then, 5 μl of each solution was spotted on TSA. After overnight incubation at 37°C, growth inhibition was observed. Samples with antibacterial activity were subjected to HPLC chromatography using an Octadecyl C18 column. After equilibrating the column with 0.1% TFA water, the sample was injected. Thereafter, a linear gradient of 0 to 60% acetonitrile for 30 min was applied to the column. Each peak was fractionated, and the samples were evaporated, then dissolved with 50 μl of distilled water. Subsequently, the antibacterial activity of each fraction was tested with the method above. ESI-MS analysis was performed by LTQ Orbitrap XL (Thermo Fisher Scientific, USA).

### Isolation of the strain curing bacteriocin-encoded plasmid

Plasmid deletion in KSE56 and KSE650 was performed with the method described elsewhere [36]. Overnight cultures of KSE56 or KSE650 were inoculated into 5 ml of fresh TSB and incubated at 37°C with shaking. When the OD660 reached 0.5, acriflavine was added at a concentration of 25 μg/ml. After incubation for 12 h, the culture was diluted and plated on TSA. After 24 h of incubation at 37°C, colonies were picked, replated on TSA and then incubated at 37°C for 24 h. Next, 0.75% soft agar (3.5 ml) containing *Bacillus coagulans* (200 μl of overnight culture) was poured on that plate and incubated at 37°C for 24 h. The strains with no inhibitory zone were picked. Finally, PCR was performed using specific primers for *S*. *epidermidis-*specific genes and bacteriocin genes coding for nukacin KSE650 or epidermin.

### Susceptibility tests

Two methods were used for the evaluation of bacteriocins. A direct assay was performed with a previously described method [34]. An overnight culture of the bacteriocin-producing strain was spotted on a TSA plate and cultured at 37°C for 24 h. Then, 3.5 ml of prewarmed half-strength TSB soft agar (1%) containing indicator bacterial cells (10^7^ cells/ml) was poured over the TSA plate. The plates were incubated at 37°C for 16 h. The diameters of the growth inhibition zones surrounding the bacteriocin-producing strains were measured in three directions. Three independent experiments were performed, and the average diameter was calculated.

Another method was to evaluate the minimal antibacterial dose of purified bacteriocins. Purified epidermin and nukacin KSE650 were adjusted to 0.5 mg/ml. The bacteriocin solution underwent 2-fold serial dilutions (2-fold to 128-fold dilution). Then, 3.5 ml of prewarmed half-strength TSB soft agar (1%) containing bacterial cells (10^7^ cells/ml) was poured over the TSA plate. Thereafter, 2 μl of the bacteriocin solutions with serial dilution (1 μg to 0.03 μg) were spotted on the plate. After the incubation at 37°C for 16 h, the minimum antibacterial dose for the growth inhibition zones was determined.

### Co-culture of *S*. *epidermidis* with *M*. *luteus*

For analysis of the proportion of each bacterium (*S*. *epidermidis* and *M*. *luteus*) in co-culture by qPCR, we first set up the method for the calculation of bacterial cell number by qPCR. A single overnight culture of the bacterium was first adjusted to OD660 = 1.0, and then a 10-fold serial dilution was performed in 500 μl of lysis buffer. After heating at 95°C for 15 min, samples were centrifuged at 15,000 x rpm for 10 min. Using the supernatant, qPCR was performed with the respective specific primers. For *S*. *epidermidis*, the forward and reverse primers used were GGCAAATTTGTGGGTCAAGA and TGGCTAATGGTTTGTCACCA, respectively. For *M*. *luteus*, the forward and reverse primers were GGGTTGCGATACTGTGAGGT and TTCGGGTGTTACCGACTTTC, respectively. Finally, the linear relationship between bacterial cell number and cut off value (Ct value) was constructed in each bacterium. Overnight cultures of *S*. *epidermidis* KSE1 (no bacteriocin production), KSE56, KSE650 and *M*. *luteus* were adjusted to OD660 = 1.0, and the bacterial culture was diluted to 10-fold. Next, 100 μl of *S*. *epidermidis* culture and *M*. *luteus* were mixed thoroughly. A small portion (20 μl) of mixed culture was spotted on TSA. After overnight incubation at 37°C, the bacterial colonies growing on agar plates were scraped and suspended in 500 μl of lysis buffer. After heating at 95°C for 15 min, the bacterial suspension was centrifuged at 15,000 x rpm for 10 min and the culture supernatant was stocked as the template for quantitative PCR (qPCR). qPCR was performed using appropriate specific primers to determine the cell number of each bacterium in the co-culture samples. Finally, the proportion of 2 bacterial species was determined. Three independent experiments were performed. Post hoc multiple comparisons were made using Tukey’s test.

## Results

### Isolation of *S*. *epidermidis* that produced bacteriocin

**Fig 1:**
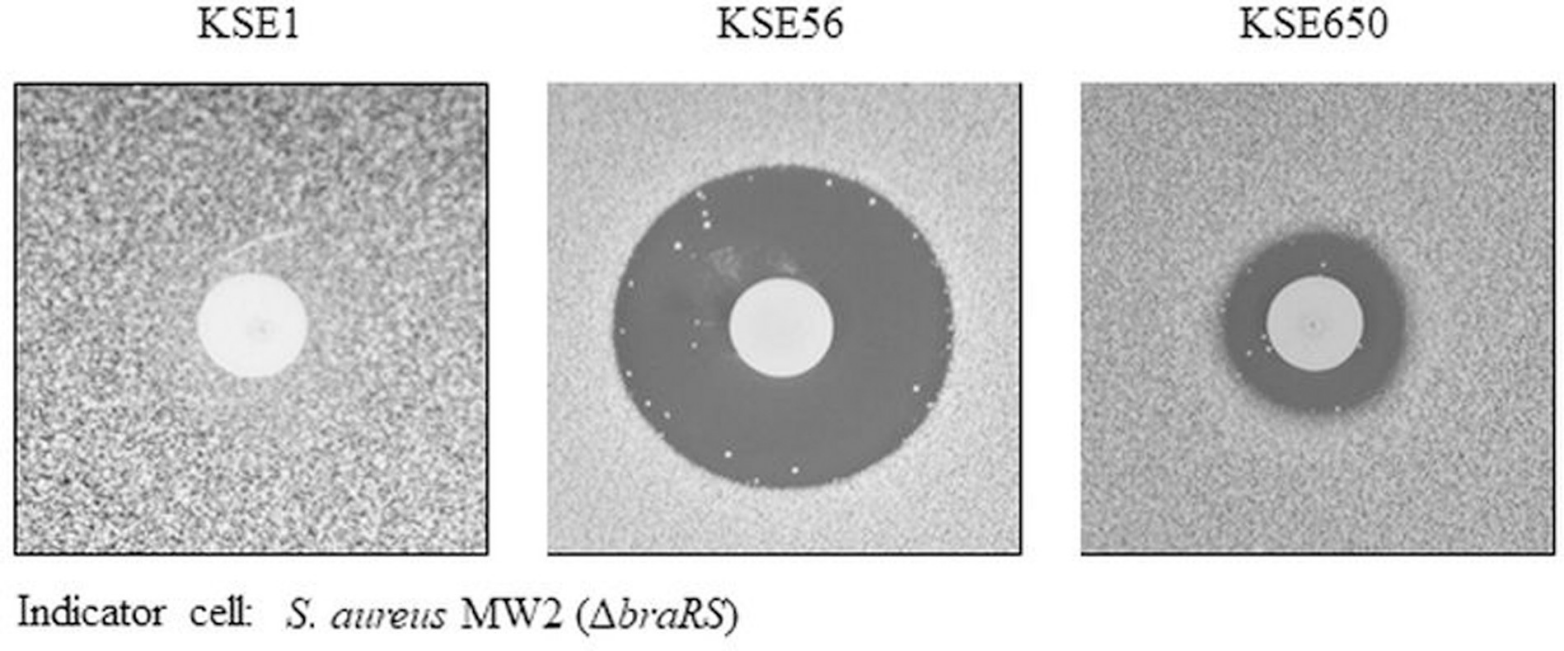
Direct assay of bacteriocin-producing *S*. *epidermidis* against *braRS*-inactivated *S*. *aureus*. The antibacterial activity of bacteriocin-producing *S*. *epidermidis* was evaluated by the direct assay using *S*. *aureus* MW2 *braRS*-inactivated mutant.

From 287 volunteers, 150 *S*. *epidermidis* strains (52.3%) were isolated from the oral cavity. Among 150 *S*. *epidermidis* strains, 2 strains showing a clear inhibitory zone against the *S*. *aureus* MW2 *braRS* inactivated mutant were identified by the direct method (Fig 1).

### Nucleotide sequence of epidermin-encoding plasmid

**Fig 2:**
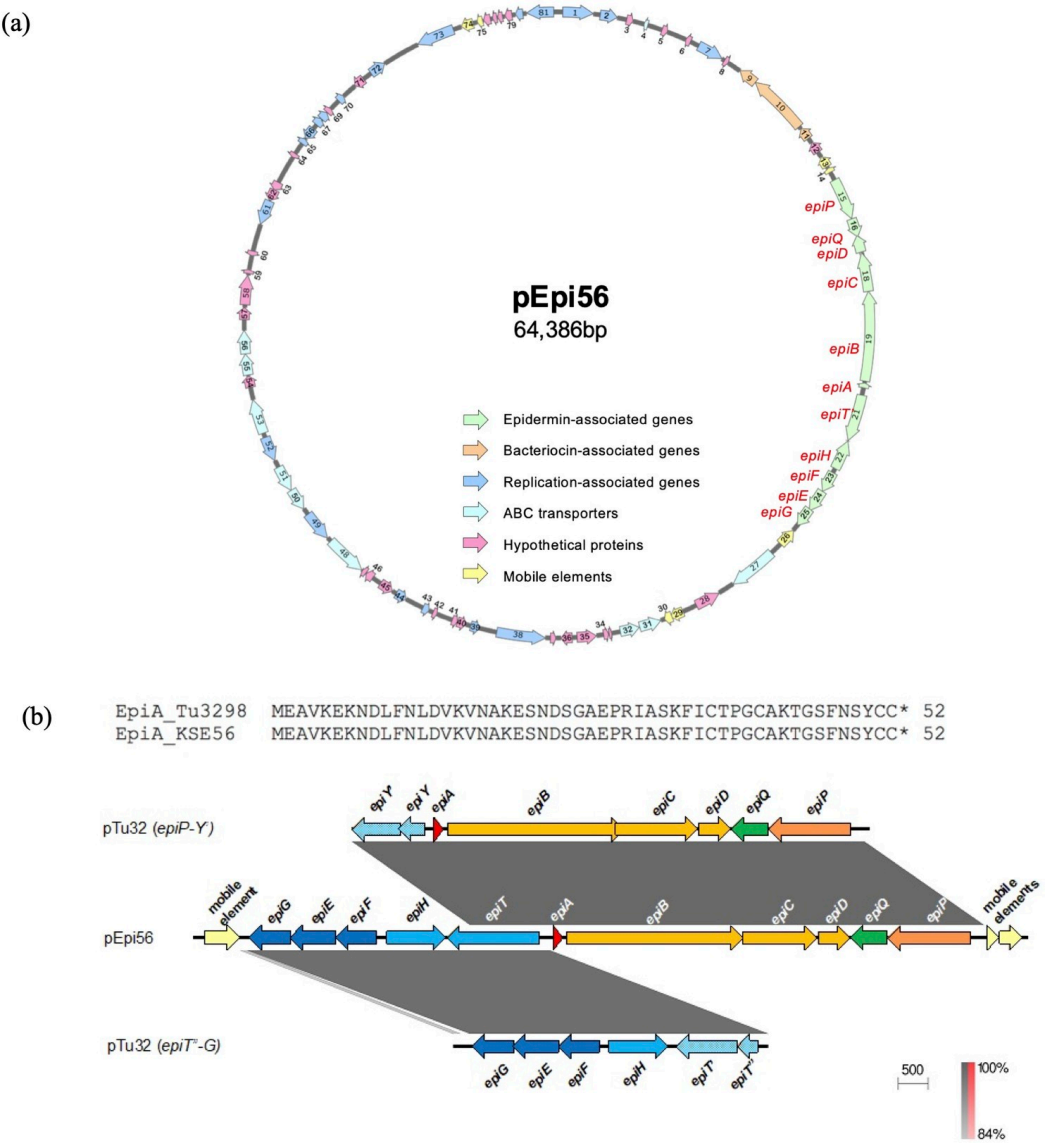
Gene map of the epidermin-carrying plasmid in KSE56. (a) Epidermin-encoding plasmid from KSE56 (pEpi56). ORFs are shown as arrows, indicating the orientation of transcription. The arrow numbers indicate the ORF number displayed in Table 2. Colors indicate the classification of gene function. (b) Bacteriocin-coding region (KSE56 epidermin). The bacteriocin-coding region from pEpi56 was compared with pTu32 *epiP-Y’* (accession number X62386) and pTu32 *epiT"-G* (accession number U77778). Striped blue arrows indicate truncated *epiT*.

The size of the entire plasmid, pEpi56, is 64,386 bp, with 81 ORFs (Fig 2A and Table 2). The plasmid contains epidermin synthesis genes (*epiA* coding for epidermin KSE56, modification genes *epiBCD*, processing genes *epiP*, export genes *epiHT*, immunity genes *epiGEF*, and regulatory gene *epiQ*), replication-related genes, and other genes including the genes coding for hypothetical proteins (Table 2). Compared with epidermin-related genes in the Tü3298 strain [19] *epiT*, which codes for an exporter, was intactin pEpi56, while a gene disrupted into two fragments (*epiT’* and *epiT”* or *epiY and epiY’*) was found in the Tü3298 strain (Figs 2B and S1). The nucleotide sequence of *epiA* in KSE56 showed 2 mismatches with that of the Tü3298 strain (S2 Fig). However, the amino acid sequence of epidermin KSE56 showed 100% identity with that in the Tü3298 strain.

**Table 2:** Genes in pEpi56.

No.	Location (bp)	Size (aa)^a^	Translation signal^b^	Homologue as determined by BLAST and/or FASTA					
				Source	Description(s)	Identity (%)	Overlap (aa)^c^	Accession no.	Note
1	190–1191	333	GAGGTTTTTTATT**ATG**	*S*. *epidermidis*	replication initiator protein A	99	333/338	WP_002498716.1	
2	1423–1983	186	AAGGAGTAATAAAA**ATG**	*S*. *epidermidis*	TIGR00730 family Rossman fold protein	99	186/186	WP_158171994.1	
3	2300–2515	71	-	*S*. *epidermidis*	hypothetical protein	67	48/78	MBM0824966.1	
4	2889–3014	41	GGAGAATAATTAATAAACCCGTTACAAAATAAGCAATATCTATAAGTTTTTTAAAAATTAAAAATTCTAAAATATGTAAGT**ATG**	*S*. *epidermidis SK135*	ATP-binding cassette domain-containing protein	100	41/41	EFA87131.1	
5	3507–3695	62	GAGTTAGACCAATAAATTGAAACGAAAAAACAATTG**TTG**	*S*. *epidermidis*	hypothetical protein	100	62/62	MBC8789835.1	
6	4346–4513	55	GGAGGCATTTGTC**ATG**	*S*. *epidermidis*	hypothetical protein	100	55/55	WP_002498713.1	
7	4819–5685	288	GGAGTGATATAT**ATG**	*S*. *epidermidis*	RepB family plasmid replication initiator protein	99	287/288	WP_203085279.1	
8	5791–5934	47	GGAGACATAAAAAGTT**ATG**	*S*. *epidermidis*	hypothetical protein	100	47/47	WP_002498711.1	
9	6397–7026	209	GAGTAATC**ATG**	*S*. *epidermidis*	ABC transporter, ATP-binding protein	100	209/209	EJD97739.1	
10	7029–9071	680	AGGTATTTATACAT**ATG**	*S*. *epidermidis NIHLM040*	bacteriocin-associated integral membrane protein	100	680/680	EJD97738.1	
11	9165–9557	130	GGAGGATTAAGTTG**ATG**	*S*. *epidermidis NIHLM040*	bacteriocin, lactococcin 972 family	100	130/130	EJD97736.1	
12	9743–10105	120	GAGAATTATACAAAA**ATG**	*S*. *epidermidis*	DUF3139 domain-containing protein	100	120/120	WP_002498706.1	
13	10304–10669	121	GAGGGACATACATTAGATATTTGG**TTG**	*S*. *epidermidis NIHLM040*	IS431mec, transposase	100	121/121	EJD97734.1	
14	10732–10884	50	GGAGTCTTCTGT**ATG**	*S*. *epidermidis NIHLM040*	hypothetical protein	100	50/50	EJD97733.1	
15	11171–12556	461	GAGGTGCTAT**ATG**	*S*. *epidermidis NIHLM040*	putative epidermin leader peptide-processing serine protease EpiP	100	461/461	EJD97732.1	*epiP*
16	12567–13184	205	GGAATAAA**ATG**	*S*. *epidermidis*	winged helix family transcriptional regulator	100	205/205	MBM0752529.1	*epiQ*
17	13181–13726	181	GGAGGAATAAGAT**ATG**	*S*. *epidermidis NIHLM040*	epidermin decarboxylase	100	181/181	EJD97730.1	*epiD*
18	13742–14992	416	GGATGGTT**GTG**	*S*. *epidermidis NIHLM040*	putative epidermin biosynthesis protein EpiC	100	416/416	EJD97729.1	*epiC*
19	14985–17945	986	GAGGTGAAATAGAA**TTG**	*S*. *epidermidis NIHLM040*	thiopeptide-type bacteriocin biosynthesis domain protein	100	986/986	EJD97728.1	*epiB*
20	18011–18169	52	AGGAGTGTTTAAA**ATG**	*S*. *epidermidis NIHLM040*	lantibiotic epidermin	100	52/52	EJD97726.1	*epiA*
21	18419–19969	516	GGACTAATATTGAGT**TTG**	*S*. *epidermidis*	ABC transporter ATP-binding protein/permease	100	516/516	WP_002498696.1	*epiT’*
22	19985–20977	330	GAGATAAGGGAGATAT**ATG**	*S*. *epidermidis*	YdcF family protein	100	330/330	WP_032605946.1	*epiH*
23	21136–21831	231	GGAGGAATAATTC**TTG**	*S*. *epidermidis*	lantibiotic protection ABC transporter ATP-binding protein	100	231/231	WP_002498693.1	*epiF*
24	21833–22597	254	GGAAATAAT**ATG**	*S*. *epidermidis*	lantibiotic immunity ABC transporter MutE/EpiE family permease subunit	100	254/254	WP_002498692.1	*epiE*
25	22587–23279	230	GGAATATAA**ATG**	*S*. *epidermidis*	epidermin immunity protein F	100	230/230	WP_002498691.1	*epiG*
26	23432–24034	200	GAGGTGGAAATCA**ATG**	*S*. *epidermidis NIHLM040*	putative transposon DNA-invertase Bin3	100	200/200	EJD97719.1	
27	24455–26071	538	GGAGGAAGAAAA**ATG**	*S*. *epidermidis NIHLM040*	ABC transporter, ATP-binding protein	100	538/538	EJD97718.1	
28	26621–27463	280	GGAGCATTAATT**ATG**	*S*. *epidermidis*	hypothetical protein	100	280/280	WP_002498688.1	
29	27952–28383	143	AAGGAGTCTTCTGT**ATG**	*S*. *epidermidis NIHLM040*	IS431mec, transposase family protein	100	143/143	EJD97715.1	
30	28376–28627	83	AGGCACCTTCAACGAAGGTAGCA**ATG**	*S*. *epidermidis NIHLM040*	IS431mec, transposase family protein	100	83/83	EJD97714.1	
31	28733–29455	240	GGAGTGTAAGCT**TTG**	*S*. *epidermidis*	peptide ABC transporter permease	100	240/240	WP_002498749.1	
32	29472–30107	211	GGAGCTGTAAACA**TTG**	*S*. *epidermidis NIHLM040*	ABC transporter, ATP-binding protein	100	211/211	EJD97793.1	
33	30389–30484	31	GGAGAGATTAA**ATG**	*S*. *epidermidis NIHLM040*	hypothetical protein	100	31/31	EJD97792.1	
34	30495–30665	56	AGGTTAATTTT**ATG**	*S*. *epidermidis*	hypothetical protein	100	56/56	TID00490.1	
35	30897–31535	212	AGGTTCAAGATGAAAACAAAGAA**ATG**	*S*. *epidermidis NIHLM040*	hypothetical protein	100	212/212	EJD97791.1	
36	31698–32063	121	GAGGAGAGAACTTTTAAA**ATG**	*S*. *epidermidis NIHLM040*	hypothetical protein	100	121/121	EJD97790.1	
37	32230–32406	58	GGAGTGATTTA**ATG**	*S*. *epidermidis NIHLM040*	hypothetical protein	100	58/58	EJD97789.1	
38	32573–34183	536	GGAAGGATTATT**ATG**	*S*. *epidermidis*	DNA mismatch repair protein MutS	100	536/536	WP_002498743.1	
39	34762–35058	98	GGATTGA**ATG**	*S*. *epidermidis*	replication initiation protein	100	98/98	MBF2337202.1	
40	35232–35510	92	GGAGAGATTAA**ATG**	*S*. *epidermidis*	hypothetical protein	100	92/92	WP_002498740.1	
41	35521–35691	56	GGATTTT**ATG**	*S*. *epidermidis*	hypothetical protein	100	56/56	WP_099800689.1	
42	36232–36369	45	GGAG ACATAAGAAGGT**ATG**	*S*. *epidermidis*	hypothetical protein	100	45/45	MBM6015004.1	
43	36517–36732	71	GGAAATGACACATCTTAAATCGACATATTCCAAAAATATGTTTAGAATACTGGTTAC**ATG**	*S*. *epidermidis*	hypothetical protein	100	71/71	WP_002498738.1	
44	37358–37726	122	GAGACGTCT**ATG**	*S*. *epidermidis NIHLM040*	hypothetical protein	100	122/122	EJD97781.1	
45	37880–38335	151	-	*S*. *epidermidis*	putative plasmid recombination enzyme	100	151/151	TID00443.1	
46	38651–38905	84	GGAGTTCCTTTAA**ATG**	*S*. *epidermidis*	hypothetical protein	100	84/84	EJD97779.1	
47	38927–39067	46	GGAAGATGAAATAGTCCTA**ATG**	*S*. *epidermidis*	hypothetical protein	100	46/46	WP_151520775.1	
48	39102–40481	459	GGAGGTATGATAG**ATG**	*S*. *epidermidis NIHLM040*	drug resistance MFS transporter, drug:H+ antiporter-2 family	100	459/459	EJD97777.1	
49	40630–41637	335	GGAGCGATGGAA**ATG**	*S*. *epidermidis*	tryptophan—tRNA ligase	100	335/335	WP_002498732.1	
50	41862–42590	242	AAGGAGAATAAACA**ATG**	*S*. *epidermidis NIHLM040*	ABC transporter permease	100	242/242	EJD97775.1	
51	42594–43457	287	AAGGAGAATAAA**ATG**	*S*. *epidermidis NIHLM040*	ABC transporter, ATP-binding protein	100	287/287	EJD97774.1	
52	43704–44525	273	GGAGGATTTT**ATG**	*S*. *epidermidis NIHLM040*	transcriptional regulator, LysR family	100	273/273	EJD97773.1	
53	44678–45817	379	GAGGATGGGATAATA**ATG**	*S*. *epidermidis NIHLM040*	MFS transporter	100	379/379	EJD97772.1	
54	46236–46613	125	GGAAAAGAGTAA**ATG**	*S*. *epidermidis NIHLM040*	hypothetical protein	96	125/125	EJE04311.1	
55	46649–47338	229	GGAGACGATAAT**GTG**	*S*. *epidermidis NIHLM040*	ABC transporter, ATP-binding protein	100	229/229	EJD97770.1	
56	47346–48107	253	GGAGGAATGAAGCAATT**ATG**	*S*. *epidermidis*	ABC transporter permease	99	253/253	WP_002503830.1	
57	48465–48857	130	-	*S*. *epidermidis NIHLM040*	hypothetical protein	100	130/130	EJD97768.1	
58	48948–49919	323	GGAGAAATT**ATG**	*S*. *epidermidis*	DUF418 domain-containing protein	99	323/323	WP_095694513.1	
59	49974–50108	44	GGAAGGA**TTG**	*S*. *epidermidis*	hypothetical protein	100	44/44	EFA87101.1	
60	50567–50722	51	-	*S*. *epidermidis*	hypothetical protein	100	51/51	MBC2926404.1	
61	51633–52454	273	AGGTGTGATTTAA**ATG**	*S*. *epidermidis*	relaxase MobL	99	273/273	WP_161382396.1	
62	52466–52849	127	GGAGGAATAAA**ATG**	*S*. *epidermidis NIHLM040*	hypothetical protein	100	127/127	EJD97765.1	
63	52851–53129	92	GGAATGATTTTT**TTG**	*S*. *epidermidis NIHLM040*	hypothetical protein	100	92/92	EJD97764.1	
64	54078–54224	48		*S*. *epidermidis*	hypothetical protein	100	48/48	WP_002456268.1	
65	54621–54800	59	GGAGGCTTATAC**ATG**	*S*. *epidermidis NIHLM040*	CsbD family protein	100	59/59	EJD97762.1	
66	54833–55231	132	GAGGTGTTTGTAT**ATG**	*S*. *epidermidis*	YolD-like family protein	100	132/132	WP_002498728.1	
67	55394–55651	85	-	*S*. *epidermidis NIHLM040*	prevent-host-death family protein	100	85/85	EJD97760.1	
68	55651–55917	88	-	*S*. *epidermidis NIHLM040*	addiction module toxin, Txe/YoeB family	100	88/88	EJD97759.1	
69	55934–56104	56	GGAGGACTCGTTA**ATG**	*S*. *epidermidis*	hypothetical protein	100	56/56	KAB2267008.1	
70	56465–56689	74		*S*. *epidermidis*	putative glycoside hydrolase	100	74/74	QRX38739.1	
71	57190–57546	118	GGAGGTTGTATGT**ATG**	*S*. *epidermidis NIHLM040*	hypothetical protein	100	118/118	EJD97756.1	
72	57860–58408	182	-	*S*. *epidermidis NIHLM040*	putative resolvase	100	182/182	EJD97755.1	
73	59658–60926	422	GGAGAATTTAATA**ATG**	*S*. *epidermidis*	penicillin-binding protein PBP4	99	422/422	WP_002498725.1	
74	61202–61603	133	-	*S*. *epidermidis*	transposase DNA-binding domain protein	100	133/133	TID00494.1	
75	61744–61926	60	GAGTCGTTTAG**ATG**	*S*. *epidermidis*	transposase	98	60/60	WP_203079065.1	
76	61958–62188	76	GAGGTGTATTGAC**ATG**	*S*. *epidermidis NIHLM040*	hypothetical protein	99	76/76	EJD97751.1	
77	62255–62407	50	GGAGGAATTAAA**TTG**	*S*. *epidermidis NIHLM040*	hypothetical protein	100	50/50	EJD97750.1	
78	62434–62595	53	GGAGGCGGGAAA**TTG**	*S*. *epidermidis*	BH0509 family protein	100	53/53	EJD97749.1	
79	62670–62909	79	GGAGGAAGATA**ATG**	*S*. *epidermidis*	hypothetical protein	100	79/79	WP_002498719.1	
80	63024–63272	82	GGAGGTATCAAGGTT**ATG**	*S*. *epidermidis*	CopG family transcriptional regulator	100	82/82	MBM0752797.1	
81	63390–64280	296	-	*S*. *epidermidis*	ParA family protein	100	268/296	WP_002498717.1	

^a^ aa, amino acids.

^b^ Bold letters indicate start codons. Underlines indicate putative ribosome binding sites complementary to the 3’ end of the 16s rRNA.

^c^ Overlap is indicated as the number of overlapping amino acids/total number of amino acids.

### Nucleotide sequence of nukacin-encoding plasmid

**Fig 3:**
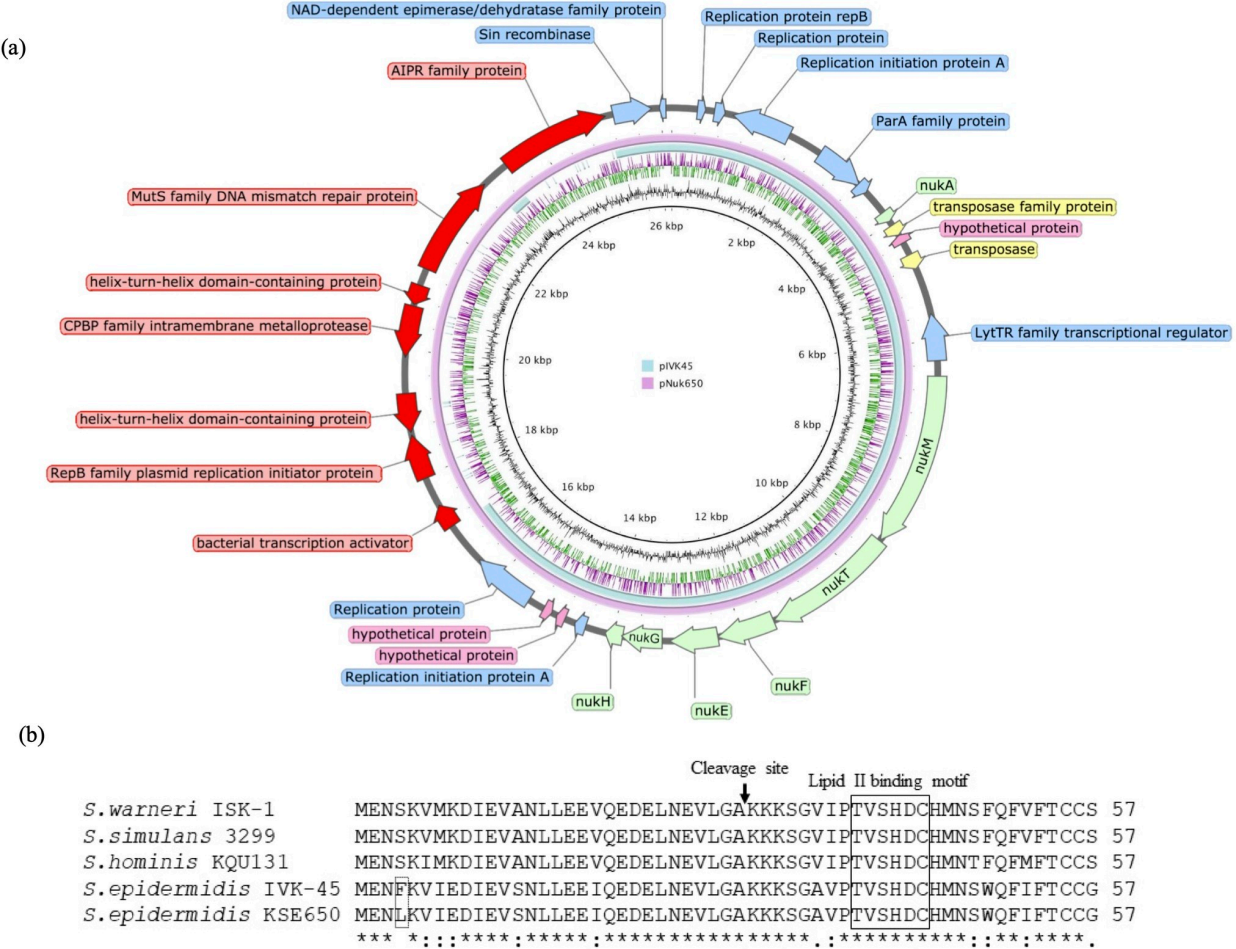
Nukacin-carrying plasmids and amino acid sequences of nukacin. (a) Nukacin-encoding plasmid from KSE650 (pNuk650) and the comparison with pIVK45. (b) Amino acid alignment of nukacin ISK-1, nukacin 3299, nukacin KQU131, nukacin IVK45 and nukacin KSE650.

The size of the entire plasmid, pNuk650, was 26,160 bp, with 29 open reading frames (ORFs). The plasmid contained nukacin KSE650 synthesis genes (*nukA* coding for prepeptide nukacin KSE650, posttranslational modification enzyme genes *nukM*, processing and secretion transporter genes *nukT*, and immunity protein genes *nukFEGH*), replication-related genes, and other genes including genes coding for hypothetical proteins (Fig 3A and Table 3). Compared to the plasmid pIVK45 (21,840 bp), which carried the gene coding for nukacin IVK45 [28] pNuk650 was larger with a higher number of ORFs (Fig 3A). The amino acid sequence of nukacin KSE650 showed similarity to nukacin IVK45 with one mismatch at the 4^th^ position, but displayed lower similarity to nukacin ISK-1 with 10 mismatches [36, 37] (Fig 3B). The mature peptide of nukacin KSE650 showed a perfect match with nukacin IVK45 and 5 mismatches with nukacin ISK-1.

**Table 3:** Genes in pNuk650.

No.	Location (bp)	Size (aa)^a^	Translation signal^b^	Homologue as determined by BLAST and/or FASTA				
				Source	Description(s)	Identity (%)	Overlap (aa)^c^	Accession no.
1	413–541	42	GGAAAAGATATCC**ATG**	*S*. *epidermidis*	RepB (pAQZ2)	83	42/42	AZL87916
2	680–850	56	-	*S*. *epidermidis*	replication protein	91	56/56	WP_194376762
3	976–1911	311	GGAAGAGGTTTATATT**ATG**	*S*. *epidermidis*	replication initiator protein A	100	311/311	WP_194378689
4	2467–3261	264	AGGAGGTATTATT**TTG**	*S*. *epidermidis*	ParA family protein	100	264/264	WP_172686110
5	3258–3467	69	GAGGGTGT**GTG**	*S*. *epidermidis*	plasmid replication associated protein, putative transcriptional regulator	98	66/69	AKQ51589
6	3821–3994	57	AGGGGGTATTATA**ATG**	*S*. *epidermidis (*pIVK45)	NukA	98	57/57	AKQ51579
7	4068–4250	60	AGGTACGCGTTTTTAAATTGTATAT**ATG**	*S*. *epidermidis*	transposase family protein	92	38/60	MBV5159007
8	4256–4393	45	GAGACC**ATG**	*S*. *epidermidis*	hypothetical protein	100	45/45	WP_194378692
9	4605–4844	79	-	*S*. *epidermidis*	transposase	100	74/79	WP_172686114
10	5583–6326	247	GAGTGAATTAT**ATG**	*S*. *epidermidis*	LytTR family transcriptional regulator DNA-binding domain-containing protein	100	247/247	WP_194378694
11	6570–9323	917	AGGAGAGGTTGTTATAT**ATG**	*S*. *epidermidis (*pIVK45)	NukM	100	917/917	AKQ51580
12	9345–11429	694	AGGTGAATACAA**TTG**	*S*. *epidermidis (*pIVK45)	NukT	99	694/694	KP702950
13	11442–12350	302	AGGAGGTTCAATTT**ATG**		NukF	99	302/302	AKQ51583
14	12351–13103	250	GGAAAGGAATATTTATAA**ATG**	*S*. *epidermidis (*pIVK45)	NukE	99	250/250	AKQ51582
15	13100–13837	245	AAGGAGAGATTTATC**TTG**	*S*. *epidermidis (*pIVK45)	NukG	88	245/245	AKQ51591
16	13844–14122	92	GAGGATTAATAACTA**ATG**	*S*. *epidermidis (*pIVK45)	NukH	100	92/92	AKQ51584
17	14444–14623	59	-	*S*. *epidermidis*	replication initiator protein A, partial	88	59/272	WP_064595943
18	14790–14930	46	GGATAACAAAATAACATCAACACAATGTCACGATTTCATAATATAGC**ATG**	*S*. *epidermidis*	hypothetical protein	98	46/46	WP_172686106
19	15014–15157	47	GGAATGATAAATTCAACTTTTTCTTTCCGATCATTAATAAAATAA**ATG**		no significant similarity found			
20	15425–16423	332	TAAGGTGTCGAATCTAAATAAAACTGGGGGCTTTTTT**ATG**	*S*. *epidermidis*	protein rep	98	332/332	WP_145461985
21	17100–17483	127	AGGGGTTTTTTT**ATG**	*S*. *epidermidis* IS-K	bacterial transcription activator, effector-binding domain protein	99	127/127	EID36019
22	17957–18664	235	GAGAGGTGTTTTTTTATGTCTGGTGAAACAGTAGTATATAGAA**ATG**	*S*. *epidermidis*	RepB family plasmid replication initiator protein	100	235/235	WP_194378685
23	18712–19323	203	AGGAGTAGTTT**ATG**	*S*. *epidermidis*	helix-turn-helix domain-containing protein	99	203/203	WP_194378686
24	19890–20699	269	GGAGAGAAATATATA**TTG**	*S*. *epidermidis*	CPBP family intramembrane metalloprotease	100	269/269	WP_168429436
25	20725–21039	104	GAGGTGTAAAAA**ATG**	*S*. *epidermidis*	helix-turn-helix domain-containing protein	99	104/104	WP_002455864
26	21312–22928	538	AGGATTATT**ATG**	*S*. *epidermidis*	MutS family DNA mismatch repair protein	99	538/538	WP_194378687
27	23374–25119	581	AGGTGAAGTTAAAA**GTG**	*S*. *epidermidis*	AIPR family protein	100	581/581	WP_194378688
28	25145–25853	202	GGAATCA**ATG**	*S*. *epidermidis (*pIVK45)	Sin recombinase	100	202/202	AKQ51586
29	25976–26077	33	AAGGAGGAATACT**ATG**	*S*. *epidermidis*	NAD-dependent epimerase/dehydratase family protein	100	33/33	WP_172686124

^a^ aa, amino acids.

^b^ Bold letters indicate start codons. Underlines indicate putative ribosome binding sites complementary to the 3’ end of the 16s rRNA.

^c^ Overlap is indicated as the number of overlapping amino acids/total number of amino acid.

### Identification of epidermin KSE56 and nukacin KSE650

**Fig 4:**
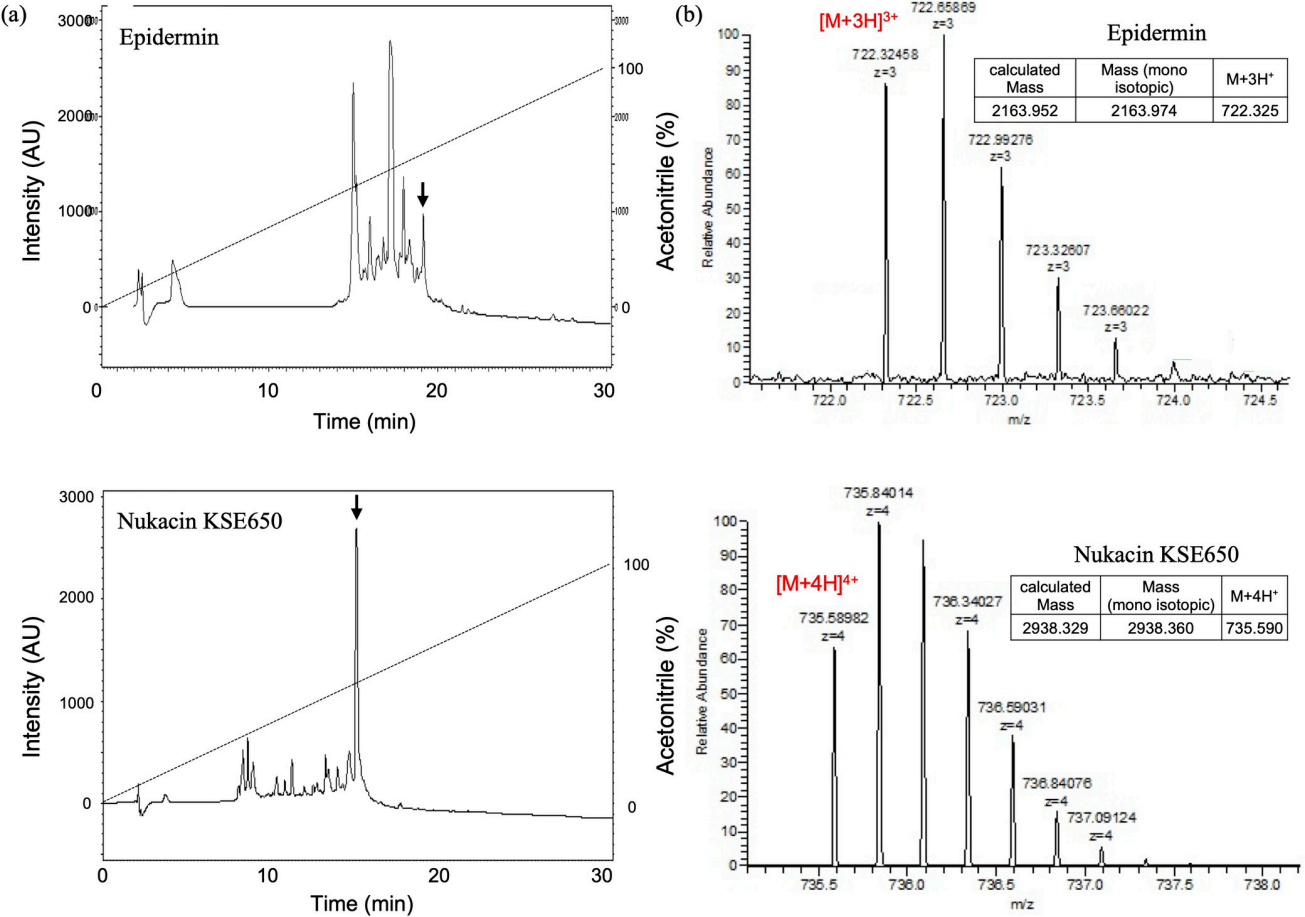
Purification of epidermin and nukacin KSE650 by reverse phase-HPLC and mass determination by ESI-MS. (a) RP-HPLC chromatogram of epidermin and nukacin KSE650. The arrow shows the peak corresponding to epidermin (upper) or nukacin KSE650 (lower). (b) Mass determination of epidermin (upper) or nukacin KSE650 (lower) by ESI-MS. Several isotopic peaks in each mass/charge (m/z) state.

Epidermin KSE56 and nukacin KSE650 were purified from the culture supernatant of KSE56 and KSE650, respectively. After applying the sample purified by Macro Prep resin to Octadecyl C18 column, peak fractions in both samples were collected and each peak fraction was checked for the antibacterial activity against *M*. *luteus*. In both samples, one peak fraction showed a strong antibacterial activity (Fig 4A). Using ESI-MS analysis, the molecular masses of purified epidermin KSE56 and nukacin KSE650 were found to be 2163.97 Da and 2938.36 Da, respectively (Fig 4B). The mass of these peptides corresponded to calculated mass of epidermin (2163.95 Da) and nukacin KSE650 (2938.33 Da).

### Antibacterial activity of epidermin KSE56 and nukacin KSE650 against several skin and oral commensal bacteria

In this study, *S*. *epidermidis* strains were isolated from the oral cavity. *S*. *epidermidis* is also known as a commensal bacterium. Therefore, we investigated the antibacterial activity of the two bacteriocins against oral and skin commensal bacterial species.

**Fig 5:**
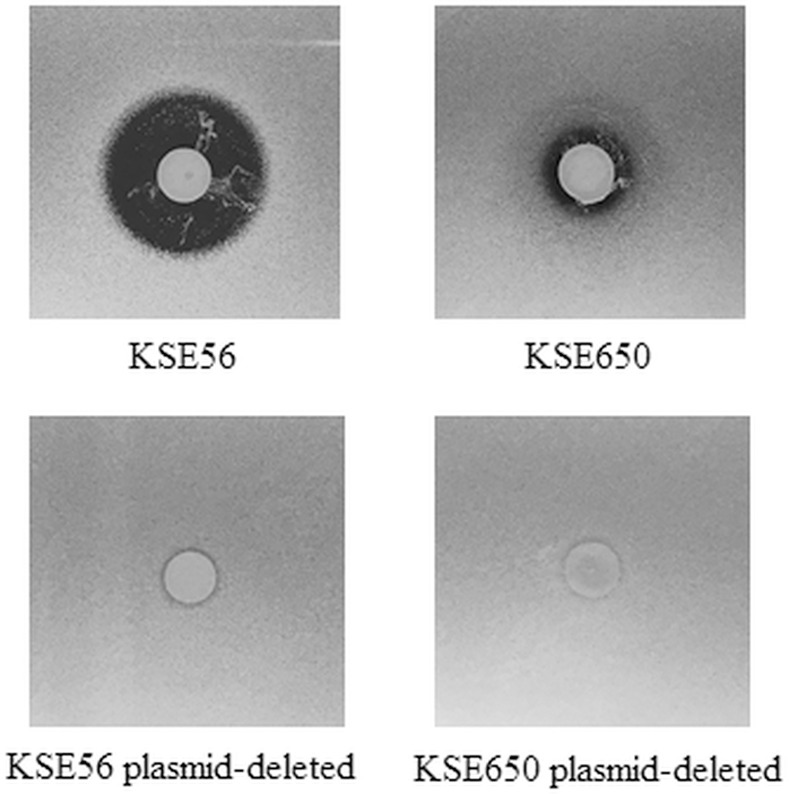
Antibacterial activity of KSE56, KSE650, and their plasmid-deleted strains. Direct assays were performed using KSE56, KSE650, and their plasmid-deleted strains. *S*. *hominis* was used as an indicator strain.

We first performed a direct assay using KSE56, KSE650 and plasmid-deleted strains. The plasmid-deleted strains showed no inhibitory zone against *S*. *hominis*, while the wild-type strains, KSE56 and KSE650, displayed inhibitory zones (Fig 5).

Afterwards, we performed a direct assay using KSE56 and KSE650 as bacteriocin-producing strains (Table 4). The epidermin-producing strain, KSE56, showed a strong antibacterial activity (>20 mm diameter inhibitory zone) against *M*. *luteus*, and an activity (>5 mm diameter) against *R*. *mucilaginosa*, *C*. *pseudodiphtheriticum*, *S*. *haemolyticus*, *S*. *captis*, *S*. *hominis*, *S*. *simulans*, and *S*. *saprophyticus*. KSE56 also showed an antibacterial activity against *S*. *epidermidis* without bacteriocin production (KSE1, 10, 12, 16), plasmid-curing KSE56 and plasmid-curing KSE650. The inhibitory zone was not observed in *S*. *epidermidis* KSE56, *S*. *epidermidis* KSE650, *C*. *accolens*, *S*. *warneri* ISK-1, and *S*. *aureus* strains. Regarding oral streptococci, KSE56 showed a strong activity against *S*. *salivarius* and *S*. *gordonii*, and modest activity against *S*. *mutans* and *S*. *sanguinis*.

**Table 4:** Antibacterial activity of KSE56 and KSE650 against various bacterial species.

Indicator strains	Halo size (mm)		
	KSE56	KSE650	*S*. *warneri*
*Corynebacterium pseudodiphtheriticum* JCM1320	10.0±0.8	10.7±0.5	11.7±0.5
*Corynebacterium accolens* JCM8331	-	-	11.3±0.5
*Micrococcus luteus* JCM1464	31.7±1.2	27.0±0	33.0±0
*Rothia mucilaginosa* JCM10910	8.7±0.5	8.0±0	13.0±0
*Cutibacterium acnes* JCM6425	15.0±0.8	-	-
*Staphylococcus haemolyticus* JCM2416	11.7±0.6	13.3±0.5	16.0±0.8
*Staphylococcus capitis* JCM2420	11.3±0.6	27.3±0.5	17.3±0.5
*Staphylococcus simulans* JCM2424	13.7±0.6	28.7±0.5	22.7±0.5
*Staphylococcus saprophyticus* JCM20595	13.0±0	12.3±0.5	13.3±0.5
*Staphylococcus hominis* JCM31912	15.3±0.6	16.3±0.5	21.7±0.5
*Staphylococcus epidermidis* KSE1	12.3±0.5	7.0±0.8	N.D.^2^
*Staphylococcus epidermidis* KSE10	12.0±0	7.3±0.5	N.D.
*Staphylococcus epidermidis* KSE12	17.0±0.8	9.7±0.5	N.D.
*Staphylococcus epidermidis* KSE16	14.3±0.5	8.7±0.5	N.D.
*Staphylococcus epidermidis* KSE56	-	-	-
*Staphylococcus epidermidis* KSE650	-	-	-
*Staphylococcus epidermidis* KSE56 plasmid-deleted	20.3±0.5	11.3±0.5	N.D.
*Staphylococcus epidermidis* KSE650 plasmid-deleted	11.0±0	11.7±0.5	N.D.
*Staphylococcus warneri* ISK-1	-	-	-
*Staphylococcus aureus* MW2	-	-	11.3±0.5
*Staphylococcus aureus* COL	-	-	11.0±0
*Staphylococcus aureus* RN4220 (MSSA)	-	-	10.7±0.5
*Streptococcus mutans* UA159	15.0±0.8	-	-
*Streptococcus sanguinis* GTC217	12.0±0	-	10.3±0.9
*Streptococcus salivarius* GTC215	27.7±0.5	12.3±0.5	18.3±0.5
*Streptococcus gordonii* JCM12995	29.0±0	17.0±0	23.0±0

"-" and "N.D." represent "no inhibitory zone" and "Not determined", respectively.

The nukacin KSE650-producing strain KSE650, showed strong antibacterial activity (>20 mm diameter) against *M*. *luteus*, *S*. *captis*, and *S*. *simulans*, and an activity (>5 mm diameter) against *C*. *pseudodiphtheriticum*, *R*. *mucilaginosa*, *S*. *haemolyticus*, *S*. *hominis*, and *S*. *saprophyticus*. KSE650 also showed an antibacterial activity against *S*. *epidermidis* without bacteriocin production (KSE1, 10, 12, 16), plasmid-curing KSE56 and plasmid-curing KSE650. The inhibitory zone was not observed in *S*. *epidermidis* KSE56, *S*. *epidermidis* KSE650, *C*. *accolens*, *S*. *warneri* ISK-1, and *S*. *aureus* strains. Regarding oral streptococci, KSE650 showed activity against *S*. *salivarius* and *S*. *gordonii*, and no activity against *S*. *mutans* and *S*. *sanguinis*. Compared to the nukacin ISK-1-producing *S*. *warneri* strain, *S*. *warneri* showed stronger activity against commensal and oral bacteria except for *S*. *capitis* and *S*. *simulans*. Notably, *S*. *warneri* ISK-1 showed activity against the *S*. *aureus* strain.

We also checked the antibacterial activity using purified epidermin and nukacin KSE650 (Table 5). The antibacterial pattern against each bacterium was similar to the results of the direct assay.

**Table 5:** Minimum antibacterial dose of purified epidermin and nukacin KSE650.

Indicator strains	Minimum antibacterial dose (μg)	
	Epidermin	Nukacin KSE650
*Corynebacterium pseudodiphtheriticum* JCM1320	2	2
*Corynebacterium accolens* JCM8331	> 2	> 2
*Micrococcus luteus* JCM1464	< 0.03	< 0.03
*Rothia mucilaginosa* JCM10910	0.5	1
*Staphylococcus haemolyticus* JCM2416	0.125	0.25
*Staphylococcus capitis* JCM2420	0.125	< 0.03
*Staphylococcus simulans* JCM2424	0.125	< 0.03
*Staphylococcus saprophyticus* JCM20595	0.5	0.25
*Staphylococcus hominis* JCM31912	0.06	0.25
*Staphylococcus epidermidis* KSE1	0.5	1
*Staphylococcus aureus* MW2	2	2
*Streptococcus mutans* UA159	1	> 2
*Streptococcus sanguinis* GTC217	0.5	1
*Streptococcus salivarius* GTC215	0.06	0.25
*Streptococcus gordonii* JCM12995	0.06	0.125

### Co-culture of *S*. *epidermidis* with *M*. *luteus*

**Fig 6:**
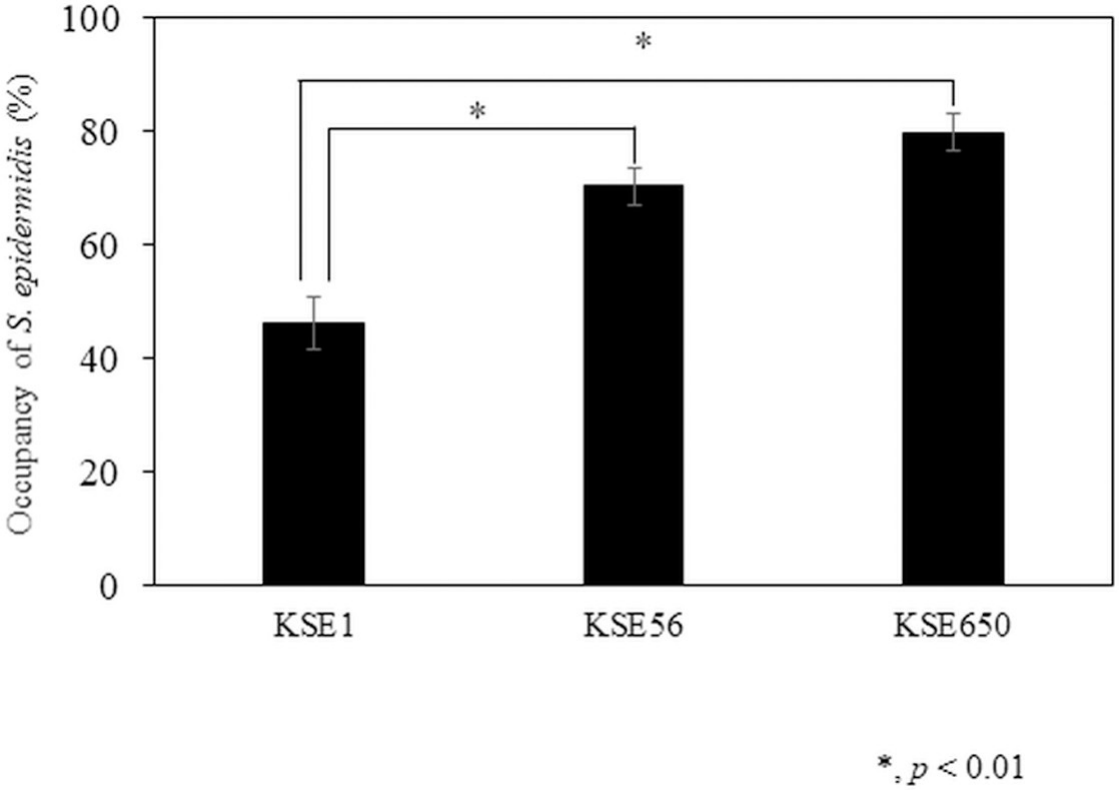
The proportion of *S*. *epidermidis* KSE1, KSE56, and KSE650 in co-culture with *M*. *luteus*. Co-culture assays were performed according to the method described in the Materials and methods. Post hoc multiple comparisons were made using Tukey’s test.

Co-cultures of *S*. *epidermidis* KSE1 (bacteriocin negative), KSE56, and KSE650 with *M*. *luteus* JCM1464 were analyzed. *M*. *luteus* was utilized as an indicator bacterium in co-culture assay because in the direct method, KSE56 and KSE650 showed a significant antibacterial effect against *M*. *luteus*. In co-culture with *M*. *luteus*, the proportion of *S*. *epidermids* KSE1 was 46.2%, while the proportions of KSE56 and KSE650 were 70.4% and 79.8%, respectively (Fig 6).

## Discussion

In this study, we tried to isolate *S*. *epidermidis* strains that produced bacteriocin. We used the *S*. *aureus* MW2 *braRS*-inactivated mutant as the indicator strain for screening. We previously reported that BraRS was involved in resistance to several bacteriocins including nisin A, nukacin ISK-1 and bacitracin [34]; therefore, a *braRS*-inactivated mutant increased susceptibility to these bacteriocins. Nisin A and nukacin ISK-1 are lantibiotics that act against lipid II molecules, which are responsible for cell wall biosynthesis, and subsequently, form a pour complex [38]. In addition, it was reported that many gram-positive bacteria, including staphylococci, streptococci, bacilli, lactococci and enterococci, produced lantibiotics that bind to lipid II [12, 19–27, 39, 40] Therefore, the *braRS*-inactivated mutant is a good indicator strain to screen lipid II-binding lantibiotics. Finally, we identified 2 strains that produce epidermin and nukacin IVK45-like bacteriocins. Whole genome analysis of the 2 strains revealed that both genes were located on the plasmids (S2A and 4 Figs).

Epidermin was first identified in the *S*. *epidermidis* Tü3298 strain [19, 41]. In the Tü3298 strain, epidermin is located on the plasmid, pTu32. Recently, the whole genome sequence of the Tü3298 strain was determined [42], but the entire plasmid sequence of pEpi56 was not reported. Therefore, our study is the first to report the complete nucleotide sequence of epidermin harboring plasmids. Additionally, the epidermin-producing strain identified in this study was the second strain, following the Tü3298 strain. The nucleotide sequence of the *epiA* coding epidermin showed 2 mismatches between the two strains, but the amino acid sequence was similar. When the epidermin synthesis genes were compared between the 2 strains, *epiT* showed a significant difference (Fig 2B). *epiT* in KSE56 was intact, while this gene in Tu3298 was disrupted into 2 genes, *epiT’* and *epiT”* in Tü3298.

EpiT is involved in the secretion of the peptide. In previous reports that demonstrated the antibacterial activity of epidermin in Tü3298 [19–21], epidermin was correctly modified and secreted externally. However, Peschel A et al reported that the introduction of intact *gdmT*, encoding the secretion protein for gallidermin, which was close to epidermin in Tü3298, increased the production of epidermin in culture supernatant [43]. Therefore, the secretion activity of epiT’/T” is considered to be partial, while the intact *epiT* gene in KSE56 may be responsible for full secretion of the epidermin peptide.

Nukacin IVK-1 was first identified in *S*. *warneri* [37]. Since then, nukacin ISK-1 like bacteriocins have been identified in *S*. *epidermidis* [28], *S*. *hominis* [44], and *S*. *simulans* [45]. The amino acid sequence of KSE650 shows a high similarity with that of IVK45 by only one mismatch in the entire peptide, and 100% match with the mature peptide. Comparison of the plasmid between the two strains showed that KSE650 was larger than Tü3298, but the composition and the order of nukacin-related genes were identical (Fig 2A). The larger size of pNuk650 was due to the insertion of an approximately 8 kbp fragment, which was detected in pNuk650 but not in pIVK45 (Fig 3A, red arrows).

**Fig 7:**
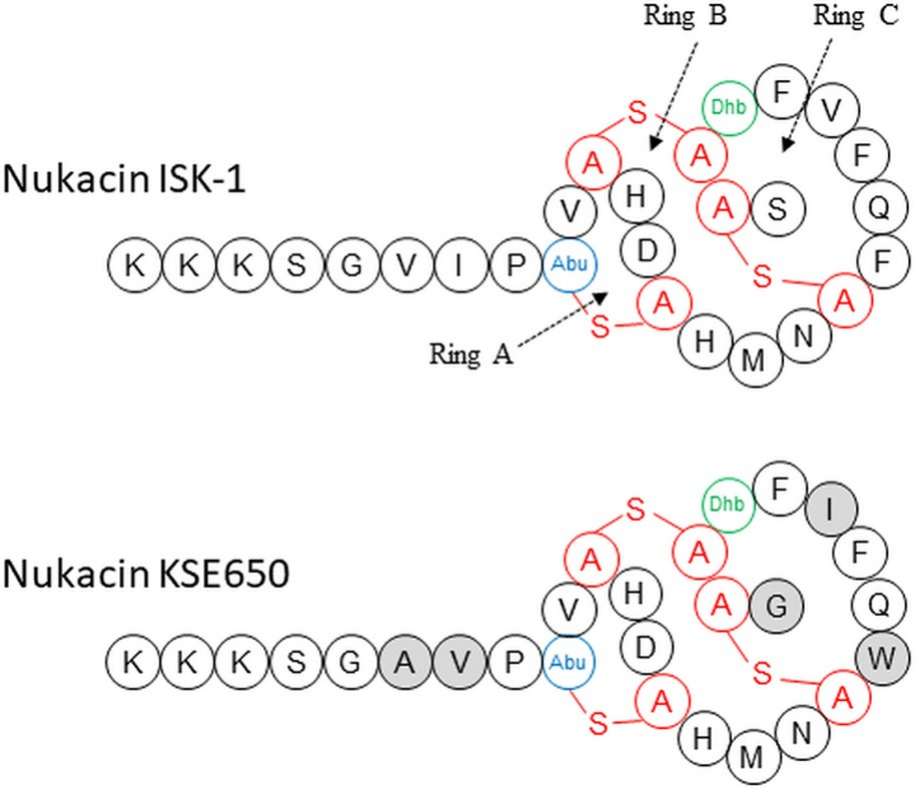
Structure of nukacin ISK-1 and nukacin KSE650. The mature peptide sequences of nukacin ISK-1 and nukacin KSE650 are shown. The deduced calculated mass of mature nukacin KSE650 is consistent with that observed by ESI-MS. The structure is identical to that of nukacin ISK-1, except for the residues indicated by gray circles. Dhb, Ala-S-Ala, and Abu-S-Ala indicate dehydrobutyrine, lanthionine, and 3-methyllanthionine respectively.

The antibacterial activity of these peptides against skin and oral commensal bacteria (oral streptococci) showed different patterns. In particular, the epidermin-producing strain (KSE56) had antibacterial activity against oral streptococci, while nukacin-producing strains had less activity. Interestingly, comparing nukacin ISK-1 and nukacin KSE650 suggested that 5 amino acid differences (Fig 7) were responsible for the different activities against several bacteria used in this study. Previously, it was reported that the structure of ring A in nukacin ISK-1 binds to the pyrophosphate moiety of lipid II, the precursor for cell wall peptidoglycan biosynthesis, and ring C was also associated with the binding of the isoprene chain [46]. Since lipid II molecules are widely conserved among gram positive bacteria, the different antibacterial activities between nukacin ISK-1 and nukacin KSE650 are influenced by the other molecules specific to each bacterial species. Furthermore, it is noteworthy that epidermin and nukacin KSE650 showed no inhibitory zone against *S*. *epidermidis* KSE650 and KSE56, respectively, while epidermin and nukacin KSE650 showed an activity against plasmid-curing KSE650 and plasmid-curing KSE56, respectively (Table 4). Although the immunity factors for epidermin and nukacin KSE650 were EpiFEG and NukFEG/NukH, respectively, which could be found in a respective plasmid, our results indicate that these immunity factors showed a cross-resistance to another bacteriocin. We previously reported that BraRS and ApsRS, TCSs, are involved in resistance to nisin A and nukacin ISK-1 [34]. Since *S*. *epidermidis* also possesses TCSs with similarity to BraRS and ApsRS, *S*. *epidermidis* TCSs may be involved in the resistance to epidermin and nukacin KSE650.

In conclusion, we determined the complete sequence of two plasmids encoding epidermin and nukacin KSE650 in *S*. *epidermidis* isolated from the oral cavity. *S*. *epidermidis* is the major commensal bacterium in human skin and the oral cavity. Based on our findings of the direct assay and co-culture assay, it is speculated that bacteriocins produced by *S*. *epidermidis* affect the bacterial composition of the host flora, including the skin, nasal and oral flora. However, in this study, we focused on the isolation of lantibiotic-producing strains using a *braRS*-inactivated strain as the indicator. Therefore, it is possible that *S*. *epidermidis* also produces other types of bacteriocins. Further studies are required to demonstrate the influence of *S*. *epidermidis* bacteriocins on the formation of bacterial flora.

## Supporting information

S1 Fig Comparison of amino acid sequences of EpiT between the KSE56 and Tü3298 strains. (PDF)

S2 Fig Comparison of nucleotide (A) and amino acid sequences (B) of epiA between the KSE56 and Tü3298 strains.
(PDF)
