# Supplementary material for: Complete sequences of epidermin and nukacin encoding plasmids from oral-derived Staphylococcus epidermidis and their antibacterial activity
Source: PLoS One. 2022 Jan 18;17(1):e0258283. doi: 10.1371/journal.pone.0258283 (PMC8765612; doi:10.1371/journal.pone.0258283)
Supplement: S1 Fig — (PDF) [file pone.0258283.s001.pdf]

```
KSE56-EpiT'      MIGTSFSLYIPLIIRNALNKSSLSTDKIVIIIICFGLTLIFSGVSTYILGYIGQKIIQNI 60
Tü3298-epiT'     ------------------------------------------------------------

KSE56-EpiT'      RSVTWNKVIKLSYSFHLKNSASNLTSRLVNDTMNITRVFSEELSTFLTNLFSVIVSLIFL 120
Tü3298-epiT'     ------------------------------------------------------------

KSE56-EpiT'      YIINKTLTLYLVCTLPILIIVILPIGNIMKRVSSKSQEATAKLSSYYSNRLSTIKLIKTL 180
Tü3298-epiT'     ------MTLYLVCTLPILIIVILPIGNIMKRVSSKSQEATAKLSSYYSNRLSTIKLIKTL 54
                       :*****************************************************
KSE56-EpiT'      STYNIEKIKNYTLLKNIFDIELHKIKVLSFFEPIMNLILFINIFGILFLGYYLMENNMMK 240
Tü3298-epiT'     STYNIEKIKNYTLLKNIFDIELHKIKVLSFFEPIMNLILFINIFGILFLGYYLMENNMMK 114
                 ************************************************************
KSE56-EpiT'      SGDMFAYVLYLFQIINPIVSITSYWTEVQRAIGSSDRILKINKEPEEVLTIKTTYNNFVQ 300
Tü3298-epiT'     SGDMFAYVLYLFQIINPIVSITSYWTEVQRAIGSSDRILKINKEPEEVLTIKTTYNNFVQ 174
                 ************************************************************
KSE56-EpiT'      KMEINDLNFRKDNKQIINSISLDLHKGYIYNIIGESGCGKSTLLNILAGLNTEYTGNICL 360
Tü3298-epiT'     KMEINDLNFTKDNKQIINSISLDLHKGYIYNIIGESGCGKSTLLNILAGLNTEYTGNIYL 234
                 ********* ************************************************ *
KSE56-EpiT'      DKLDKSQFSKYEWRNLFSYITQDLQILEDTVYNNLIYGINENISIEEIQNACKKTNSLNF 420
Tü3298-epiT'     DKLDKSQFSKYEWRNLFSYITQDLQILEDTVYNNLIYGINENISIEEIQNACKKTNSLTF 294
                 **********************************************************.*
KSE56-EpiT'      IQNLKNGFSTSISPDSINLSIGQKQRLVLTRAFLQKKPIILLDEVTSNLDKESHKYIVKS 480
Tü3298-epiT'     IQNLKNSFSTSISPDSINLSIGQKQRLVLTRAFLQKNLLFY------------------- 335
                 ******.*****************************: ::
KSE56-EpiT'      IESLAQSSIVLNVTHRHDKNDFSNSKVKLIDFRQFN 516
Tü3298-epiT'     ------------------------------------
```

Supplemental Fig. 1. Comparison of amino acid sequences of epiT between KSE56 and Tü3298 strain

```
KSE56-EpiT′     MIGTSFSLYIPLIIRNALNKSSLSTDKIVIIIICFGLTLIFSGVSTYILGYIGQKIIQNI 60
Tü3298-epiT''   MIGTSFSLYIPLIIRNALNKSSLSTDKIVIIIICFGLTLIFSGVSTYILGYIGQKIIQNI 60
                ************************************************************

KSE56-EpiT′     RSVTWNKVIKLSYSFHLKNSASNLTSRLVNDTMNITRVFSEELSTFLTNLFSVIVSLIFL 120
Tü3298-epiT''   RSVTWNKVIKLPYSFHLKNSASNLTSRLVNDTMNITRVFSVEF------IFSYSITNIFI 114
                ***********.**************************** *:      :**  :: **:

KSE56-EpiT′     YIINKTLTLYLVCTLPILIIVILPIGNIMKRVSSKSQEATAKLSSYYSNRLSTIKLIKTL 180
Tü3298-epiT''   YN---------------------------------------------------------- 116
                *

KSE56-EpiT′     STYNIEKIKNYTLLKNIFDIELHKIKVLSFFEPIMNLILFINIFGILFLGYYLMENNMMK 240
Tü3298-epiT''   ------------------------------------------------------------
KSE56-EpiT′     SGDMFAYVLYLFQIINPIVSITSYWTEVQRAIGSSDRILKINKEPEEVLTIKTTYNNFVQ 300
Tü3298-epiT''   ------------------------------------------------------------
KSE56-EpiT′     KMEINDLNFRKDNKQIINSISLDLHKGYIYNIIGESGCGKSTLLNILAGLNTEYTGNICL 360
Tü3298-epiT''   ------------------------------------------------------------
KSE56-EpiT′     DKLDKSQFSKYEWRNLFSYITQDLQILEDTVYNNLIYGINENISIEEIQNACKKTNSLNF 420
Tü3298-epiT''   ------------------------------------------------------------
KSE56-EpiT′     IQNLKNGFSTSISPDSINLSIGQKQRLVLTRAFLQKKPIILLDEVTSNLDKESHKYIVKS 480
Tü3298-epiT''   ------------------------------------------------------------
KSE56-EpiT′     IESLAQSSIVLNVTHRHDKNDFSNSKVKLIDFRQFN 516
Tü3298-epiT''   ------------------------------------
```

Supplemental Fig. 1. Comparison of amino acid sequences of epiT between KSE56 and Tü3298 strain
