# Supplementary material for: Complete sequences of epidermin and nukacin encoding plasmids from oral-derived Staphylococcus epidermidis and their antibacterial activity
Source: PLoS One. 2022 Jan 18;17(1):e0258283. doi: 10.1371/journal.pone.0258283 (PMC8765612; doi:10.1371/journal.pone.0258283)
Supplement: S2 Fig — Comparison of nucleotide (A) and amino acid sequences (B) of epiA between the KSE56 and Tü3298 strains. (PDF) [file pone.0258283.s002.pdf]

(A)

```
epiA:Tü3298 atggaagcagtaaaagaaaaaaatgatctttttaatcttgatgttaaagttaatgcaaaa 60
epiA:KSE56  atggaagcagtaaaagaaaaaaatgatctttttaaccttgatgttaaagttaatgcaaaa 60
            *********************************** ************************

epiA:Tü3298 gaatctaacgattcaggagctgaaccaagaattgctagtaaatttatatgtactcctgga 120
epiA:KSE56  gaatctaacgattcaggagctgaaccaagaattgctagtaaatttatatgtactcctgga 120
            ************************************************************

epiA:Tü3298 tgtgcaaaaacaggtagttttaacagttattgttgttaa 159
epiA:KSE56  tgtgcaaaaacaggtagttttaacagttattgctgttaa 159
            ******************************** ******
```

(B)

```
EpiA:Tü3298 MEAVKEKNDLFNLDVKVNAKESNDSGAEPRIASKFICTPGCAKTGSFNSYCC* 52
EpiA:KSE56  MEAVKEKNDLFNLDVKVNAKESNDSGAEPRIASKFICTPGCAKTGSFNSYCC* 52
            *****************************************************
```

Supplemental Fig. 2. Comparison of nucleotide (A) and amino acid sequences (B) of *epiA* between KSE56 and Tü3298 strain
